# Risk Factors for Adverse Neurodevelopment in Transient or Persistent Congenital Hyperinsulinism

**DOI:** 10.3389/fendo.2020.580642

**Published:** 2020-11-30

**Authors:** Marcia Roeper, Roschan Salimi Dafsari, Henrike Hoermann, Ertan Mayatepek, Sebastian Kummer, Thomas Meissner

**Affiliations:** Department of General Pediatrics, Neonatology and Pediatric Cardiology, University Children’s Hospital, Medical Faculty, Heinrich-Heine-University Duesseldorf, Duesseldorf, Germany

**Keywords:** brain injury, hyperinsulinism, hypoglycemia, outcome, neurodevelopment, risk factors

## Abstract

**Objective:**

Aim was to identify hypotheses why adverse neurodevelopment still occurs in children with transient or persistent hyperinsulinism despite improvements in long-term treatment options during the last decades.

**Material and Methods:**

A retrospective review of 87 children with transient (n=37) or persistent congenital hyperinsulinism (CHI) (n=50) was conducted at the University Children’s Hospital Duesseldorf, Germany. Possible risk factors for neurodevelopmental sequelae due to hypoglycemia were analyzed with a focus on the first days after onset of disease.

**Results:**

Median age at follow-up was 7 years (IQR 8). Adverse neurodevelopmental outcome was seen in 34.5% (n=30) of all CHI patients. Fifteen had mildly abnormal neurodevelopment and 15 had a severe hypoglycemic brain injury. In univariate analysis, mildly abnormal neurodevelopment was associated with the diagnosis of persistent CHI (odds ratio (OR) 8.3; p=0.004) and higher birth weight (mean difference 1049 g; p<0.001). Severe hypoglycemic brain injury was associated with the diagnosis of persistent CHI (OR 5.1; p=0.013), being born abroad (OR 18.3; p<0.001) or in a lower-level maternity hospital (OR 4.8; p=0.039), and of note history of hypoglycemic seizures (OR 13.0; p=<0.001), and a delay between first symptoms of hypoglycemia and first blood glucose measurement/initiation of treatment (OR 10.7; p<0.001). Children with severe hypoglycemic brain injury had lower recorded blood glucose (mean difference -8.34 mg/dl; p=0.022) and higher birth weight than children with normal development (mean difference 829 g; p=0.012). In multivariate binary logistic regression models, lowest blood glucose <20 mg/dl (OR 134.3; p=0.004), a delay between initial symptoms and first blood glucose measurement/initiation of treatment (OR 71.7; p=0.017) and hypoglycemic seizures (OR 12.9; p=0.008) were positively correlated with severe brain injury. Analysis showed that the odds for brain injury decreased by 15% (OR 0.85; p=0.035) if the blood glucose increased by one unit.

**Conclusion:**

While some risk factors for adverse outcome in CHI are not influenceable, others like lowest recorded blood glucose values <20 mg/dl, hypoglycemic seizures, and insufficiently—or even untreated hypoglycemia can be avoided. Future guidelines for management of neonatal hypoglycemia should address this by ensuring early identification and immediate treatment with appropriate escalation steps.

## Introduction

Congenital Hyperinsulinism (CHI) is a rare disorder characterized by inappropriately increased insulin secretion from pancreatic beta cells. It is the most common cause of persistent hypoglycemia in neonates and infants with an estimated incidence of about 1:50.000 live births (1). More than ten genes and further syndromic disorders (e.g. Beckwith-Wiedemann syndrome, Sotos syndrome, Kabuki syndrome, Rubinstein-Taybi syndrome or Turner syndrome) have been recognized to be associated with this disease (2–5). However, the underlying genetic cause is only identified in about 50%–60% of patients (6, 7). A more common cause for prolonged neonatal hypoglycemia is transient hyperinsulinism, which can be regularly seen in infants of diabetic mothers, children who are large or small for gestational age, asphyxiated newborns or late preterm babies (8, 9). Unlike persistent CHI, this condition usually resolves spontaneously within a few weeks to a few months (10).

Patients with CHI are at high risk for brain injury due to severe neuroglycopenia and suppression of alternative fuels such as ketone bodies during hyperinsulinemic hypoglycemia (11). Brain injury may occur in both transient and persistent forms of hyperinsulinism (12). The prevalence of neurodevelopmental sequelae is reported to be up to 48% in patients with persistent CHI and 30% in children with transient CHI, respectively (13, 14). However, two recent studies indicate that the prevalence of brain injury is similar in children with transient and persistent CHI (12, 15). Furthermore, no significant improvement of the neurodevelopmental outcome can be seen over the last decades despite advances in treatment options (13, 14, 16, 17). Known risk factors associated with worse neurodevelopmental outcome in children with CHI include neonatal onset of the disease, delayed diagnosis and insufficient stabilization of plasma glucose levels (18, 19). However, identification of other influenceable risk factors might allow further improvements in therapeutic or diagnostic strategies.

In this study, we examined the neurodevelopmental outcome of a cohort of children with persistent as well as transient CHI, aiming to identify risk factors associated with hypoglycemic brain damage that could be addressed by improved management to prevent neurologic sequelae in the future. The analysis focuses on data regarding the onset of disease with respect to diagnosis and adequate treatment.

## Material and Methods

Retrospective medical record review of 87 children with transient and persistent CHI treated and regularly seen at the University Children’s Hospital, Duesseldorf, Germany, between 2008 and 2019 was conducted. While 8% (n=7) were born at the University Children’s Hospital Duesseldorf, the remaining patients were either transferred to our center for continuation of inpatient treatment or they were seen at our outpatient hyperinsulinism clinic, where patient care is continued after discharge from hospital.

In a first step, data of 126 patients with CHI were screened for inclusion and exclusion criteria for the study. Inclusion criterion was the diagnosis of transient or persistent CHI, which was based on recurrent hypoketotic hypoglycemia with measurable elevated insulin levels during hypoglycemia and/or high glucose requirements >8 mg/kg/min to maintain blood glucose (BG) >50 mg/dl. Transient CHI was defined as a neonatal hyperinsulinemic condition in which hypoglycemia persisted beyond the first week of life but resolved spontaneously within 12 months of life without any further necessity for medical treatment.

Exclusion criteria were conditions that are regarded as an additional risk factor for brain injury independent of hypoglycemia: suspicion of syndromic disorders, chromosomal aberration, metabolic disease, preterm birth <34 weeks of gestation, intracranial hemorrhage and perinatal asphyxia. In addition, all children below 1 year of age at last follow-up were excluded, because it is difficult to assess the neurological development at this young age. Therefore, a total number of 39 patients were excluded from this study.

Final data of the remaining 87 patients were then analyzed retrospectively for possible risk factors for hypoglycemic brain injury with the focus on the onset of disease and the period before transfer to a specialized center. To supplement the retrospective data, a parental questionnaire was designed for this study and was administered to all families included in the study (**Supplementary Material**). The questionnaire was completed by 33 families. Median age at follow-up was 7 years [interquartile range (IQR) (8)].

Voigt et al. percentiles were used to evaluate children’s birth weight (20). Children were considered small for gestational age (SGA) when their birth weight was -2 standard deviation (SD) below the norm and large for gestational age (LGA) when their birth weight was above +2 SD from the norm. Level of maternity hospital was evaluated and divided into two groups. Lower-level hospital included basic care and specialty care nursery. High-level hospital included subspecialty care including neonatal intensive care unit (NICU) and regional perinatal center including NICU. In terms of neurological outcome, a diagnosis of epilepsy, hearing deficits, vision impairment, microcephaly and cerebral palsy were assessed in the retrospective data. The data were supplemented by information on cerebral magnetic resonance imaging (MRI) and electroencephalography, if available in the medical records. Developmental outcome was recorded if the assessment was made by a psychologist or pediatrician and according to the parental questionnaire. In Germany, all children have regular standardized check-ups at their local pediatrician including neurodevelopmental evaluation. These check-ups are recorded in the children’s examination booklet which is used to follow the children’s development from birth until adolescence. In addition, neurodevelopment is assessed at our hyperinsulinism clinic at every appointment. When these assessments show abnormalities, children are referred for neurological testing. Due to the retrospective character of this study, standardized neurodevelopmental testing was hence only performed upon suspicion of adverse neurodevelopment and age and method of testing varied accordingly. Tests conducted in the cohort included the Bayley Scale of Infant and Toddler Development—Third Edition, the Wechsler Intelligence Scale for Children—Fourth Edition, the Movement Assessment Battery for Children—Second Edition, the Snijders-Oomen Non-verbal intelligence test, and the German Developmental testing 6 months to 6 years. If there was a suspicion of a specific developmental disorder, separate and specified testing was carried out.

After data collection was completed, patients were assigned to three different groups according to their neurodevelopmental outcome: normal, mildly abnormal or severe hypoglycemic brain injury. Severe hypoglycemic brain injury was defined as a diagnosis of epilepsy, severe neurosensory impairment (including vision or hearing deficit), severe neurodevelopmental delay in more than two areas or signs of hypoglycemic brain injury in MRI scans. Mildly abnormal neurodevelopment was defined as an early onset partial neurodevelopmental delay in a maximum of two different areas with the need for sustained supportive therapy.

### Statistical Analysis

Statistical analysis was performed using SPSS^®^ Statistics (IBM SPSS Statistics. 2017. Version 25.0. Armonk, NY). Baseline data were assessed using standard descriptive statistics (percentage, mean, median, SD and IQR. Binary and multivariate comparisons between the three groups were performed. Kolmogorov-Smirnov-Test was used to test for normality of distribution of continuous variables. Student’s t-test and one-way ANOVA with post-hoc comparison were used to analyze parametric variables and Mann-Whitney-U test and Kruskal-Wallis test were applied when variables were nonparametric. Pearson’s Chi-squared test and Fisher’s Exact test were used when appropriate to analyze associations between categorical variables. Univariate and multivariate binary logistic regression models with stepwise backward logistic regression were applied to determine variables that significantly affected the outcome. Odds ratio (OR) and 95% confidence interval (CI) are reported. A p-value <0.05 was considered statistically significant.

The study was approved by the institutional review board of the Medical Faculty of the Heinrich-Heine-University Duesseldorf, Germany, and was performed in accordance with the Declaration of Helsinki.

## Results

In total, 87 patients with CHI were included in the study. 42.5% (n=37) were female. Children were born at a mean of 38 ± 2 weeks of gestation. 57.5% (n=50) had persistent CHI and 42.5% (n=37) had transient CHI. Patient characteristics are displayed in **Table 1**.

**Table 1:** Patient characteristics.

	Total	Normal development	Mildly abnormal development	Severe brain injury	p-value*
**Number of patients**	87	57	15	15	
**Age (years)**, *median (IQR)*	7 (8)	6 (9)	12 (11)	7 (5)	0.681
**Female**, *n (%)*	37 (42.5)	23 (40.4)	5 (33.3)	9 (60)	0.173
**Transient CHI**, *n (%)*	37 (42.5)	32 (56.1)	2 (13.2)	3 (20)	**0.019**
**Persistent CHI**, *n (%)*	50 (57.5)	25 (43.9)	13 (86.7)	12 (80)	**0.019**
**Birth weight (g)**, *mean ± SD*	3,222 ± 1,006 (n = 77)	2,892 ± 779 (n = 50)	3,940 ± 1,013 (n = 14)	3,720 ± 1,220 (n = 13)	**0.012**
**Percentile (Voigt)**, *median (IQR)*	27 (89) (n = 73)	13.5 (48) (n = 48)	96 (71) (n = 13)	67 (82) (n = 12)	**0.011**
**SDS (Voigt)**, *median (IQR)*	−0.6 (3.2) (n = 73)	−1.1 (1.9) (n = 48)	1.7 (4.3) (n = 13)	0.5 (3.4) (n = 12)	**0.011**
**Gestational age (weeks)**, *mean ± SD*	38 ± 2 (n = 75)	38 ± 2 (n = 48)	38 ± 2 (n = 14)	38 ± 3 (n = 13)	0.509
**Born abroad**, *n (%)*	9 (10.3)	2 (3.5)	1 (6.7)	6 (40)	**<0.001**
**Born *via* C-section**, *n (%)*	48 (68.6) (n = 70)	33 (71.7) (n = 46)	9 (76.9) (n = 13)	7 (63.6) (n = 11)	0.728
**Risk factor for neonatal hypoglycemia**, *n (%)*	44 (58.9) (n = 76)	28 (57.1) (n = 49)	10 (76.9) (n = 13)	6 (42.9) (n = 14)	0.378
**Early onset CHI (<30 days p.n.)**, *n (%)*	68 (78.2)	45 (78.9)	14 (93.3)	9 (60)	0.180
Delay between first symptoms and first BG measurement**, *n (%)*	19 (24) (n = 79)	7 (13.7) (n = 51)	3 (21.4) (n = 14)	9 (60)	**<0.001**
**Delay between first symptoms and first BG measurement (days)**, *mean ± SD*	127 ± 179 (n = 19)	119 ± 124 (n = 7)	162 ± 93 (n = 3)	123 ± 241 (n = 9)	0.363
**Pancreatic surgery**, *n (%)*	10 (11.5)	3 (5.3)	2 (13.3)	5 (41.7)	**0.008**
**Hypoglycemic seizures**, *n (%)*	37 (42.5)	19 (33.3)	5 (33.3)	13 (86.7)	**<0.001**
**Symptoms at onset of hypoglycemia**, *n (%)*	62 (78.5) (n = 79)	36 (70.6) (n = 51)	12 (85.7) (n = 14)	14 (93.3)	0.093
**Genetic CHI mutation**, *n (%)*	29 (47.5) (n = 61)	13 (40.6) (n = 32)	8 (53.3) (n = 15)	8 (57.1) (n = 14)	0.301
Birth at lower-level hospital***, *n (%)*	25 (39.7) (n = 63)	14 (32.6) (n = 43)	4 (40) (n = 10)	7 (70) (n = 10)	**0.039**

*Comparison of normal development vs. severe brain injury only. n, number; SD, standard deviation; %, percent; IQR, interquartile range; BG, blood glucose; CHI, congenital hyperinsulinism; p.n., postnatal. N-values are only described in case of missing data. Bold type indicates significant result. **Delay between first symptoms and first BG measurements included patients who had clear symptoms of hypoglycemia but had a delay >12 h between onset of symptoms and first BG measurement ***Lower-level hospital included basic care and specialty care nursery. High-level hospital included subspecialty care including neonatal intensive care unit (NICU) and regional perinatal center including NICU.

Adverse neurodevelopmental outcome was seen in 34.5% (n=30) of all patients. Of these, 15 had severe hypoglycemic brain damage and 15 had mildly abnormal neurodevelopment. The prevalence of neurodevelopmental disorders was 13.5% (n=5) in subjects with transient CHI and 50% (n=25) in patients with persistent CHI, respectively. Persistent CHI was associated with an increased risk for both mildly abnormal development (OR 8.3; CI 1.7–40.3; p=0.004) and severe brain injury (OR 5.1; CI 1.3–20.1; p=0.013). The specific presentation of abnormal neurodevelopmental outcome is shown in **Table 2**. Both gender and gestational week were not predictive for brain injury (p=0.173 and p=0.509, respectively).

**Table 2:** Neurodevelopmental outcome.

	Total (n = 87)	Mildly abnormal development (n = 15)	Severe brain injury (n = 15)
**Epilepsy**	8	–	8
**Visual impairment**	2	–	2
**Hearing deficit**	2	–	2
**Cerebral palsy**	2	–	2
**Microcephaly**	3	–	3
**Signs of brain injury in MRI scans** (n = 24)	7	–	7
**Abnormal EEG** (n = 32)	13*	3	9
**Neurodevelopmental delay**	28	15	13
**≤2 areas**	21	15	6
**≥3 areas**	7	–	7
- Speech	9	6	3
- Psychiatric/behavioral	8	5	3
- Cognition	2	0	2
- Motor	16	9	7
- Global	4	–	4
Severe mental retardation	2	–	2
- ADD/ADHD	2	2	0
- Hyperactivity/attention deficit (no AD(H)D diagnosis)	7	4	3
**Neurocognitive testing**	21	8	8

Data are presented as number. *Including one child without abnormal neurodevelopment. MRI, magnetic resonance imaging; EEG, electroencephalography; (AD(H)D), attention deficit (hyperactivity) disorder.

**Figure 1:**
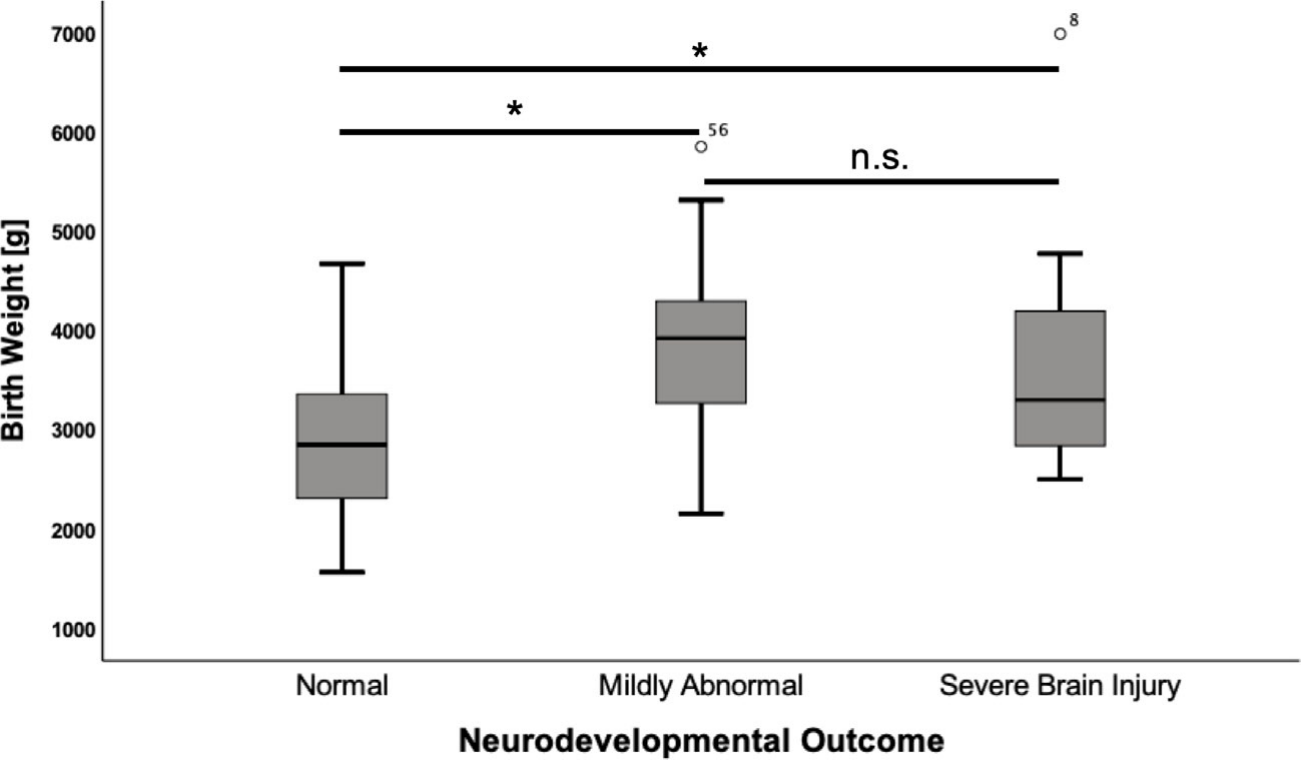
Birth weight according to neurodevelopmental outcome. Values are displayed as mean ± standard deviation. ANOVA with Tukey *post hoc* comparison showed significant differences in the mean for normal neurodevelopmental outcome (n = 50) vs. mildly abnormal development (n = 14) (2,892 ± 779 vs. 3,940 ± 1,013, p = <0.001) and normal neurodevelopmental outcome vs. severe brain injury (n = 13) (2,892 ± 779 vs. 3,720 ± 1,220, p = 0.012). *p = < 0.05. n.s., not significant.

Mean birth weight in the cohort was 3222 g (SD ± 1006). Infants who suffered from hypoglycemic brain damage had a significantly higher birth weight than those with normal development (+ 943 g; CI 513–1373; p<0.001). A correlation was noted for both mild (p<0.001) and severe (p=0.012) neurodevelopmental impairment in comparison to children with normal development (**Figure 1**).

89.6% (n=78) of patients were born in Germany and 10.3% (n=9) were born abroad. Countries of birth included Syria, Iraq, India, Turkey, Spain, Croatia, Kosovo and Macedonia. All but one foreign-born patient migrated to Germany in infancy and long-term care was continued at our center after relocation. Adverse neurodevelopment was very common in patients born abroad (77.8%) and the odds for severe brain injury were significantly increased (OR 18.3; CI 3.2–105.4; p<0.001). Separate subgroup analysis of patients born abroad is presented in **Table 6** in the **Supplementary Material**.

31.4% (n=22) of individuals were born *via* spontaneous vaginal delivery and 68.6% (n=48) were born *via* C-section. 60.8% (n=38) were born at a higher-level maternity center. While the mode of delivery had no impact on the neurodevelopmental outcome, being born at a lower-level maternity hospital was a risk factor for severe brain injury (OR 4.8; CI 1.1–21.6; p=0.039). Patients born at lower-level maternity hospitals had both higher rates of brain injury in transient CHI (25%, n=3) and in persistent CHI (61.5%, n=8) compared to patients born at higher-level hospitals, where the rates of adverse neurodevelopment were 8.7% (n=2) in transient CHI and 46.7% (n=7) in persistent CHI, respectively. However, no significant difference was shown in binary comparison using Chi-squared test or Fisher’s Exact test (transient CHI p=0.313, persistent CHI p=0.476). Furthermore, no differences were seen when comparing group characteristics of patients born at lower-level or higher-level maternity hospitals (**Table 3 Supplementary Material**). 78.9% (n=68) of patients had an early onset of hypoglycemia (< 30 days of life) while 21.8% (n=19) had a late presentation (> 30 days of life). However, there was no correlation between age at onset of hypoglycemia and neurodevelopmental outcome (p=0.807). Presence of general risk factors for neonatal hypoglycemia was common in children with CHI (58.9%, n=44). The most common risk factors included SGA (n=11), LGA (n=15), late preterm birth (n=17) and adaption disorder (n=14). There was no association between presence of a risk factor in general and abnormal neurodevelopmental outcome (p=0.378). Macrosomia was related to a mildly abnormal neurodevelopment (OR 7.4; CI 1.8–30.8; p=0.008), however, no association was found in relationship to severe hypoglycemic brain injury (p=0.069) in comparison to the normal outcome group.

24% (n=19) of subjects had a delay of more than 15 h between first observed symptoms of hypoglycemia, first BG measurement and initiation of treatment (as observed in the retrospective parents’ questionnaires and in medical record review). Mean time of delay until treatment in this group was 127 days (range 0.5–730). There was a strong correlation between delayed diagnosis/treatment and severe neurodevelopmental impairment (OR 10.7; CI 2.9–39.4; p<0.001). This correlation was also significant when differentiating between short delay (0.5–3 days) and long delay (30–730 days) (OR 41.7; CI 4.1–418.9; p<0.001 and OR 6.7; CI 1.4–31.9; p=0.026, respectively).

78.5% (n=62) of individuals presented with symptomatic hypoglycemia at onset of the disease, however, there was no association between presence or absence of symptoms in general and neurodevelopmental outcome. Of note, 42.5% (n=37) had at least one episode of a hypoglycemic seizure in their history. Presence of a hypoglycemic seizure as a symptom was significantly associated with severe brain injury (OR 13.0; CI 2.7–63.6; p<0.001).

**Figure 2:**
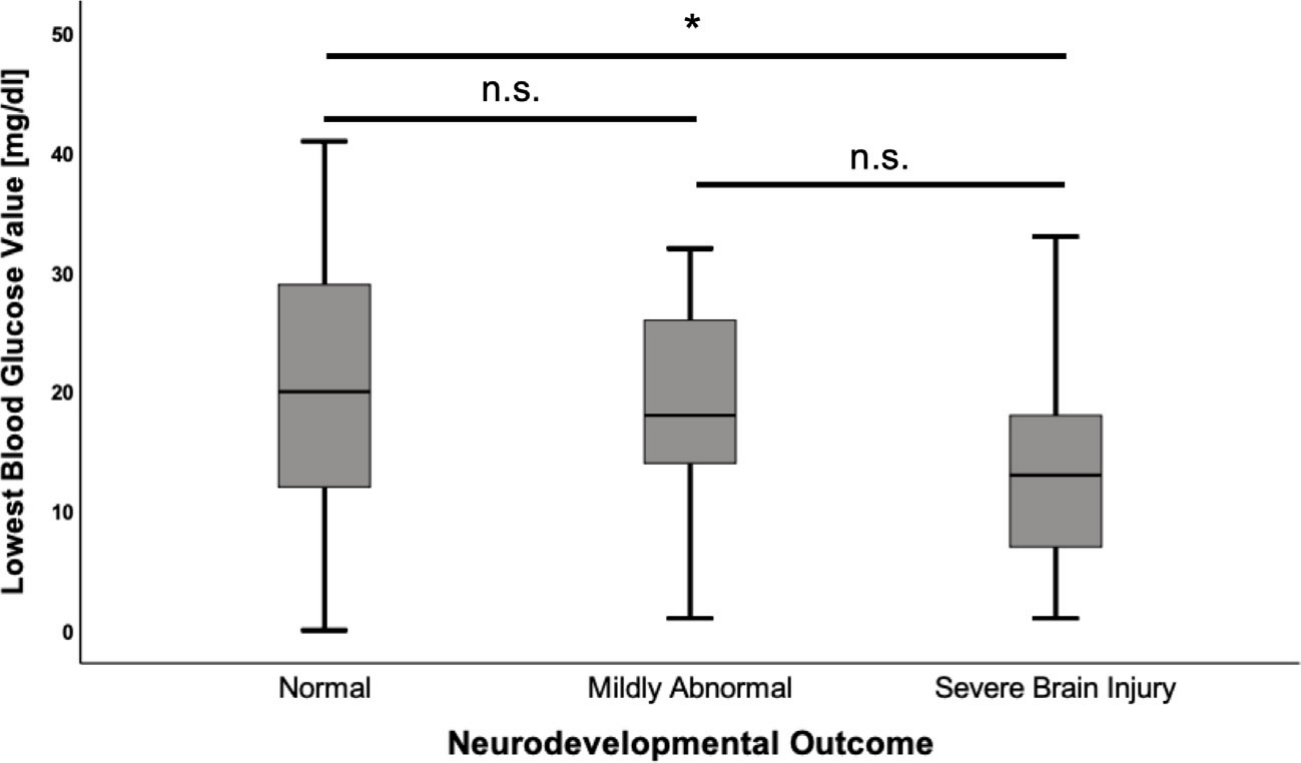
Lowest blood glucose values according to neurodevelopmental outcome. Values are displayed as mean ± standard deviation. ANOVA with Tukey *post hoc* comparison showed significant differences in the mean for normal neurodevelopmental outcome (n = 53) vs. severe brain injury (n = 14) (20.6 ± 11.0 vs. 12.3 ± 8.6, p = 0.022). Comparison between the remaining groups showed no significant differences. *p < 0.05, n.s, not significant.

Mean of first BG analysis in the cohort was 21.4 ± 13.7 mg/dl and the mean of the lowest recorded BG was 18.7 ± 10.6 mg/dl. Insulin/glucose-ratio during hypoglycemia was 1.6 ± 5.5 mU/l/mg/dl and the mean of highest glucose infusion rate was 14.5 ± 3.8 mg/kg/min. There was no correlation between first BG analysis, insulin/glucose-ratio or highest glucose infusion rate and the neurodevelopmental outcome (all p-values >0.05). However, the mean of lowest BG was significantly lower in patients with severe brain injury compared to children with regular development (-8.34 mg/dl; CI -15.69–0.98; p=0.022) (**Figure 2**), and significantly more children in the severe brain injury group had a lowest recorded BG < 20 mg/dl (85.7% vs. 47.2%; OR 6.7; CI 1.4–33.0; p=0.014). Differences in laboratory results between the different outcome groups are shown in **Table 3**.

**Table 3:** Laboratory measurements and treatment.

	Total	Normal development	Mildly abnormal development	Severe brain injury	p-value*
**First BG (mg/dl)**, *mean* ± *SD*	21.4 ± 13.7 (n = 64)	22.8 ± 12.7 (n = 40)	24.0 ± 16.3 (n = 12)	14.1 ± 12.7 (n = 12)	0.051
**Lowest BG (mg/dl)**, *mean* ± *SD*	18.7 ± 10.6 (n = 81)	20.6 ± 11.0 (n = 53)	18.1 ± 8.6 (n = 14)	12.3 ± 8.6 (n = 14)	**0.022**
**Lowest BG <20 mg/dl,** *n (%)*	45 (55.6) (n = 81)	25 (47.2) (n = 53)	8 (57.1) (n = 14)	12 (85.7) (n = 14)	**0.014**
**Insulin during hypoglycemia (mU/L)**, *median (IQR)*	12.5 (22) (n = 79)	13.1 (21) (n = 51)	15.9 (16) (n = 15)	6.3 (24) (n = 13)	0.659
**Diazoxide-unresponsive**, *n (%)*	14 (18.2) (n = 77)	6 (11.8) (n = 51)	4 (28.6) (n = 14)	4 (33.3) (n = 12)	0.086
**Max. i.v. glucose infusion rate (mg/kg/min)**, *mean* ± *SD*	14.5 ± 3.8 (n = 47)	13.9 ± 4.0 (n = 29)	15.4 ± 3.2 (n = 10)	15.4 ± 3.6 (n = 8)	0.328
**Insulin/glucose ratio (mU/l/mg/dl),** *mean* ± *SD*	1.6 ± 5.5 (n = 72)	1.3 ± 5.0 (n = 44)	1.0 ± 1.2 (n = 15)	3.4 ± 8.9 (n = 13)	0.888

*Comparison of normal development vs. severe brain injury only. Bold type indicates significant result. n, number; SD, standard deviation; %, percent; IQR, interquartile range; BG, blood glucose; i.v., intravenous.

Regarding treatment of CHI, 18.2% (n=14) of patients did not respond to diazoxide. Pancreatic surgery was performed in 11.5% (n=10) of patients with persistent CHI and there was a significant association with severe neurodevelopmental sequelae in this severely affected cohort (OR 9.0; CI 1.9–43.8; p=0.008). Genetic testing was carried out in 70.1% (n=61) of patients. The standard primary genetic panel for hyperinsulinism at our clinic includes the following genes: ABCC8, GCK, GLUD1, HADH, HK1, HNF1A, HNF4A, INSR, KCNJ11, PMM2, SLC16A1, UCP2 (since 2015 also KDM6A and KMT2D). A genetic mutations associated with CHI was found in 47.5% (n=29) of cases (90% (n=26) in those with persisting CHI, 10% (n=3) in those with transient CHI). The most commonly diagnosed genetic mutations were pathogenic K_ATP_ channel variants (*ABCC8* and *KCJN11*) in 20 patients. However, finding of such a variant was not associated with worse neurodevelopmental outcome (p=0.731). Two infants had hyperinsulinism-hyperammonemia syndrome due to a GLUD1 mutation but did not suffer any brain injury. Genetic analysis of the cohort is displayed in **Tables 1** and **2** of the **Supplementary Material**.

In a multivariate binary logistic regression model with backward elimination of all Germany-born patients including all significant risk factors for adverse neurodevelopmental outcome from the prior univariate analysis, only birth weight had a trend towards an increased risk for mildly abnormal development (OR 1.001; CI 1.0–1.002; p=0.002). Lowest BG < 20 mg/dl (OR 134.3; CI 1.3–14361; p=0.004) and a delay between initial symptoms and first BG measurements, as well as initiation of treatment (OR 71.7; CI 2.2–2379; p=0.017), were positively correlated with severe brain injury. When lowest BG < 20 mg/dl was substituted for lowest recorded BG in mg/dl in this model, it was also significant. Analysis showed that the odds for brain injury decreased by 15% (OR 0.85; CI 0.73–0.98; p=0.035) if the BG increased by one unit. In a second model for severe brain injury, including the remaining risk factors that did not show any positive correlation in the first model, occurrence of hypoglycemic seizures was significantly associated with severe brain injury (OR 12.9; CI 1.9–86.4; p=0.008). The regression models are displayed in **Table 4**.

**Table 4:** Multivariate binary logistic regression analysis (Germany-born patients only).

	Model 1 (n = 59) Normal vs. mildly abnormal development			Model 2 (n = 46) Normal vs. severe brain injury			Model 3 (n = 48) Normal vs. severe brain injury		
**Variables**	Odds ratio	95% CI	p-value	Odds ratio	95% CI	p-value	Odds ratio	95% CI	p-value
**Diagnosis (transient vs. persistent CHI)**	n.s.			n.s			n.s.		
**Birth weight (g)**	1.001	1.0–1.002	0.002	n.s.			n.s.		
**Delay between first symptoms and first BG measurement**	E			71.7	2.2–2379	0.017	E		
**Pancreatic surgery**	E			n.s.			n.s.		
**Hypoglycemic seizures**	E			n.s.			12.9	1.9–86.4	0.008
**Birth at lower-level hospital**	E			n.s.			n.s.		
**Lowest BG < 20 mg/dl**	E			134.3	1.3–14361	0.004	E		
**Lowest BG (mg/dl)**	E			0.85*	0.73–0.98	0.035	E		

Only significant variables from univariate analysis were entered into the multivariate logistic regression models. Patients born abroad were excluded from the analysis due to the high rate of adverse neurodevelopment in this small subgroup. Data are presented as odds ratio for adverse neurodevelopment with 95% confidence interval and p-value, if significant <0.05.

BG, blood glucose; CHI, congenital hyperinsulinism; OR, odds ratio; CI, confidence interval; n.s., not significant; E, excluded in model.

Model 1: included the following risk factors: diagnosis, birth weight, percentile. Backward logistic regression was performed.

Model 2: included the following risk factors: diagnosis, birth weight, delay between first symptoms and first BG measurement, pancreatic surgery, hypoglycemic seizures, birth at lower-level hospital, lowest BG < 20 mg/dl. Backward logistic regression was performed. *When lowest BG < 20 mg/dl is exchanged for lowest BG value in mg/dl (metric variable), the result is also significant.

Model 3: included the following risk factors: diagnosis, birth weight, pancreatic surgery, hypoglycemic seizures, birth at lower-level hospital. Backward logistic regression was performed.

## Discussion

In this study, we retrospectively analyzed risk factors associated with an adverse neurodevelopmental outcome in children with transient and persistent CHI to identify approaches to improve the neurological prognosis of affected patients in the future.

In our cohort, adverse neurodevelopmental outcome was observed in 50% of patients with persistent CHI and 13.5% of children with transient CHI, respectively. This result is in line with other studies reporting neurodevelopmental sequelae in 25%–48% of patients with persistent CHI (13–19, 21). Data concerning the outcome after transient CHI are still limited. In 2013, a study by Avatapalle et al. found abnormal neurodevelopment in 30% of children with transient CHI and reported no significant differences regarding the incidence of neurodevelopmental difficulties compared to children with persistent CHI (12). Correspondingly, a recent Finnish study found similar rates of neurodevelopmental impairment in both groups (25% in persistent CHI and 27% in transient CHI, respectively). In contrast, data from Japan revealed that the prevalence of developmental delay and epilepsy was significantly higher in patients with persistent CHI (28.0% and 14.2%) than in patients with transient CHI (11.7% and 2.0%) (22). Our study also showed that patients with persistent CHI had significantly higher odds for both mildly abnormal development (OR 8.3) and severe brain injury (OR 5.1) compared to patients with transient CHI. Even though patients with these both forms of the disease might present similar during the neonatal period, management and stabilization of BG are often more complicated in patients with persistent CHI, especially if they have K_ATP_ channel mutations. Yet, mild neurodevelopmental sequelae of neonatal hypoglycemia might not be detected until (pre-) school age (23, 24). Therefore, adverse outcome in some children with transient CHI might remain undiagnosed since standardized neurodevelopmental testing is not regularly performed in these children after the condition has resolved. Still, our results show that also severe brain injury can occur after transient CHI and adverse neurodevelopment is frequent in children with transient CHI being born at lower-level maternity hospitals. Thus, neurological consequences should not be underestimated in these patients. We strongly recommend that children with all forms of CHI should receive standardized neurodevelopmental testing at different ages to detect any abnormal development at an early stage and initiate supportive treatment, if necessary.

Like Helleskov et al. (14), we could not confirm a difference between neonatal-onset or infantile-onset of hypoglycemia in terms of hypoglycemic brain injury, as it has been previously described (12, 16, 18, 21, 25). However, the distribution of children with early or late onset of CHI was quite disproportionate in our analysis, so the result should be interpreted with caution.

In 2003, Meissner et al. described a higher birth weight in children with neonatal onset of CHI but did not find any difference regarding the neurodevelopmental outcome (16). In our study, increased birth weight was significantly correlated with neurodevelopmental impairment. However, as increased birth weight indicates prenatal hyperinsulinism and increased levels of insulin promote fetal anabolism, it may simply reflect disease severity and thus associate with adverse neurodevelopmental outcome. In this regard, infants with macrosomia and neonatal hypoglycemia should be closely monitored and congenital hyperinsulinism should be ruled out if hypoglycemia persists beyond the first 48 h of life.

In general, it is difficult to objectively quantify the severity of CHI, as there is no common consensus on criteria, thus making a comparison of results between different studies difficult (12, 14). In our analysis, we estimated and quantified disease severity by highest intravenous (i.v.) glucose demand, insulin/glucose-ratio and presence of a K_ATP_ channel mutation, although this choice of parameters is highly arbitrary. There was no correlation found for either of these factors with the neurodevelopmental outcome. However, we found that severe neurodevelopmental impairment was more prevalent in surgically than in medically treated patients, as it has previously been described by different authors (18, 21, 26). One explanation may be that patients requiring pancreatectomy often have pathogenic K_ATP_ channel variants and simply reflect the most severe neonatal presentation of hypoglycemia (21). In addition, the expression of defective K_ATP_ channels in brain tissue could potentially influence brain function and development (27). In our study, most surgically treated patients also presented pathogenic variants in K_ATP_ channel genes. Nevertheless, we found no association between the neurodevelopmental outcome and CHI genetics, as is has been previously reported by other studies (13, 14, 17). However, the sample size for gene variants other than K_ATP_ was too small to perform subgroup analysis. Further studies in larger cohorts of patients with different mutations causing CHI are needed to fully understand which impact the genetic diagnosis may have on the neurodevelopmental outcome.

Besides the diagnosis of persistent CHI, the strongest associations between risk factors and severe brain injury in multivariate analysis were found for hypoglycemic seizures, lowest recorded BG < 20 mg/dl and history of probably untreated hypoglycemia, indicated by a delay between initial symptoms, first recorded BG measurement and initiation of adequate treatment. We speculate that at least a subgroup of patients has been symptomatic before the glucose drops to a level less than 20 mg/dl or before patients present with a hypoglycemia seizure. In univariate analysis, children born abroad, mainly in developing countries were also at increased risk for neurodevelopmental sequelae. It seems likely that these children received at least in part suboptimal treatment due to limited resources in medical care or limited experience with management of severe hypoglycemia. Furthermore, birth at a lower-level maternity hospital was associated with higher odds for severe brain injury in univariate analysis. Although sufficient medical resources should be available in all German hospitals, we hypothesize that these poorer outcomes are mostly due to limited experience with symptoms and management of severe hypoglycemia and timely delay until the child is evaluated by a pediatrician and transferred to a NICU for adequate i.v. glucose treatment. Albeit there is a national guideline for treatment of neonatal hypoglycemia in Germany, it officially applies only to infants of diabetic mothers and treatment recommendations for severe and recurrent hypoglycemia are missing. It therefore provides limited advice for health care professionals who are inexperienced in the treatment of severe hypoglycemia (28). We therefore promote both improvement of national guidelines as well as better education about neonatal hypoglycemia to prevent brain injury in children affected by severe hypoglycemia.

Helleskov et al. have also advocated education of health care professionals regarding neonatal hypoglycemia and CHI as one potential preventive measure (14). The authors reported an association between neurodevelopmental impairment and lowest recorded BG < 18 mg/dl, birth abroad (non-Scandinavian) as well as a delay from first symptoms to expert treatment center. They concluded that insufficiently- or untreated hypoglycemia was the major determinant of brain injury in CHI, and proposed that spread of knowledge about CHI and prompt contact with an expert center in case of suspicion of CHI are mandatory to improve the outcome in patients with CHI (14).

The risk factors for brain injury identified here are linked in several ways and must not be regarded as independent risk factors: un- or insufficiently treated hypoglycemia regularly results in low BG exchange levels for concentrations and these in turn may lead to hypoglycemic seizures due to neuroglycopenia. Especially in children with CHI, low blood sugars pose an extreme risk for brain injury as alternative energy sources i.e. ketone bodies or free fatty acids are simultaneously suppressed. Nevertheless, developmental delay has also been reported after transient and mild neonatal hypoglycemia. In 2015, Kaiser et al. related poorer proficiency on fourth-grade literacy and mathematics tests to early transient neonatal hypoglycemia (24). A prospective cohort study revealed an association of neonatal hypoglycemia and an increased risk of reduced executive—and visual-motor function at 4.5 years (23). Furthermore, the authors detected severe, recurrent or undetected hypoglycemia as a risk factor for an unfavorable outcome. Thus, data indicating harm from neonatal hypoglycemia are increasing.

It has to be stated that there are also some limitations in this study. First, the design is observative and retrospective. Hence, a variable number of data were missing and analysis of potential risk factors is prone to underreporting, at least in some of the participants. Second, there might have been some selection bias as children with missing follow-up until at least 1 year of age were excluded from the study. Third, no standardized psychometric testing was performed in all patients, thus reducing the quality of data. Therefore, we cannot rule out that especially mild developmental delay has not been diagnosed in some children or an impairment might not have been recognized by parents in the questionnaire. This applies particularly for children with transient CHI as data on follow-up after recovery from the condition was often limited. In general, the length of the follow-up period varied, so longitudinal developmental data are not available for some patients. However, since data on neurodevelopmental impairment is much more likely to be underestimated than overestimated, our results are all the more important and call for timely improvement in management of neonatal hypoglycemia and CHI. Lastly, even though we analyzed a relatively large cohort given the rarity of the disease, the sample size does not allow detailed subgroup analyses, making an interpretation difficult.

Despite these limitations, our study revealed several risk factors for severe brain injury in patients with CHI. Further research and prospective longitudinal studies are needed to fully understand the mechanisms and risk factors which still lead to brain injury in a considerable number of patients with CHI.

## Conclusion

Despite improvement in treatment options, neurodevelopmental sequelae are still worryingly frequent in children with CHI. Our study revealed risk factors for the development of hypoglycemic brain injury in affected patients. While risk factors like a diagnosis of persistent CHI, pancreatic surgery, birth abroad or an increased birth weight are hardly influenceable, others like lowest recorded BG values < 20 mg/dl, hypoglycemic seizures, and insufficiently—or untreated hypoglycemia are avertable determining factors for severe brain damage related to CHI. Our results indicate, that particularly management of neonatal hypoglycemia during the first days of life is crucial for the later outcome. Education and awareness of health care professionals about neonatal hypoglycemia and CHI as well as national guidelines on neonatal hypoglycemia need to be improved to facilitate early recognition and adequate treatment of affected patients. Standardized neurodevelopmental testing should be performed in all children with persistent and transient CHI at different ages and developmental stages to ensure early recognition of adverse outcome to provide supportive treatment.

## Data Availability Statement

The raw data supporting the conclusions of this article will be made available by the authors, without undue reservation.

## Ethics Statement

The studies involving human participants were reviewed and approved by the institutional review board of the Medical Faculty of the Heinrich-Heine-University Duesseldorf, Germany. Written informed consent from the participants’ legal guardian/next of kin was not required to participate in this study in accordance with the national legislation and the institutional requirements.

## Author Contributions

MR designed the study, created the questionnaire, collected and interpreted the data, and wrote the initial manuscript. RSD contributed to the study design and creation of the questionnaire and revision and review of the manuscript. HH collected and interpreted the data and critically revised and reviewed the manuscript. EM interpreted and critically validated the data and revised and reviewed the manuscript. SK provided expertise as a pediatric endocrinologist and specialist for congenital hyperinsulinism and neonatal hypoglycemia, critically validated the data, and revised and reviewed the manuscript. TM contributed to designing the study, creating the questionnaire, and writing the initial manuscript. All authors contributed to the article and approved the submitted version.

## Funding

Financial support for this study was provided by the Ilse-Bagel Foundation.

## Conflict of Interest

The authors declare that the research was conducted in the absence of any commercial or financial relationships that could be construed as a potential conflict of interest.

## Abbreviations

BG, blood glucose; CHI, congenital hyperinsulinism; CI, 95% confidence interval; i.v., intravenous; IQR, interquartile range; LGA, large for gestational age; MRI, magnetic resonance imaging; OR, odds ratio; SD, standard deviation; SGA, small for gestational age.
