# Supplementary material for: Risk Factors for Adverse Neurodevelopment in Transient or Persistent Congenital Hyperinsulinism
Source: Front Endocrinol (Lausanne). 2020 Nov 30;11:580642. doi: 10.3389/fendo.2020.580642 (PMC7793856; doi:10.3389/fendo.2020.580642)
Supplement: Supplementary file 1 [file DataSheet_1.docx]

Supplementary Material

**Table 1: Genetic analysis**

|  | **Total** | **Normal**  **development** | **Mildly abnormal**  **development** | **Severe brain injury** | **p-value*** |
| --- | --- | --- | --- | --- | --- |
| **ABCC8** | 15 | 6 | 5 | 4 | 0.465 |
| **KCJN11** | 5 | 3 | 1 | 1 |  |
| **HADH** | 3 | 2 | 0 | 1 |  |
| **GCK** | 1 | 0 | 1 | 0 |  |
| **GLUT1** | 2 | 2 | 0 | 0 |  |
| **others** | 4 | 1 | 1 | 2 |  |
| **No result** | 32 | 19 | 7 | 6 |  |
| **Not performed** | 26 | 25 | 0 | 1 |  |
| **K_ATP_** | 20 | 9 | 6 | 5 | 0.731 |

Data are presented as number. One child had a mutation in both ABCC8 and HADH. * Comparison of normal development vs. severe brain injury only.

**Table 2: Genotype profiles in the cohort**

| **ABCC8 Variants:** homozygous c.4612C>T (p.Arg1538*)  homozygous c.4612C>T (p.Arg1538*)  homozygous missense-mutation c.563A>G (p.N188Ser)  compound heterozygous c.1183A>T, c.4146T>G  compound heterozygous c.50T>C (p.Val17AIa), c.3653+1G>A; p.=?)  compound heterozygous mutation in Exon 12 and Intron 15  heterozygous maternal inherited IVS9-A c.1468-2A>C Acceptor splice site  heterozygous c.1252T>C (p.Cys418Arg)  heterozygous c.4516G>A  heterozygous (Exon 39c 4.613 G>A)  heterozygous c.1176+2T>C (IVS7+2T>C)  heterozygous c.4374G>C (p.Gin1458His) in Exon 36  heterozygous c.4516G>A (p.(Glu1506Lys)  heterozygous Exon 29c.3640>Tp.Arg1214Trp  heterozygous paternal inherited variant (c.2506C>T, p.Arg836*)  **GLUD1 Variants:** heterozygous c-965 ARG>HIS heterozygous c.808T>A | **KCJN11 Variants:**  heterozygous c.526C>T  homozygous c.405dupG (pARG136Alafs*)  heterozygous missense-mutation (c.118G>A, p.G40S)  heterozygous paternal inherited mutation c.391A>G (p.Ile131Val)  heterozygous c.11G>A, p.R4H  **HADH Variants:**  heterozygous c.275T>G (p.Phe92Cys)  homozygous C.428T>G (p.Ile143Asn)  homozygous C.428T>G (p.Ile143Asn)  **GCK Variants:** missing data for exact variant  **Others:**  heterozygous SLC16A1 (c.1063G>A, p.Gly355Arg in Exon 4)  heterozygous INSR (c.1238 G>A P.Arg 413His)  heterozygous CACNA1C (c.1697T>C) p.Leu566Pro  HNF4a (missing data for exact variant) |
| --- | --- |

**Table 3: Comparison of patient characteristics according to level of maternity hospital*****

|  | **Patients born at lower-level maternity hospital** | **Patients born at higher-level maternity hospital** | **p-value*** |
| --- | --- | --- | --- |
| **Number of patients** | 25 | 38 |  |
| **Age [years],** *median (IQR)* | 7 (6) | 5 (7) | 0.083 |
| **Female,** *n (%)* | 10 (40) | 16 (42.1) | 1.0 |
| **Transient CHI,** *n (%)* | 12 (48) | 23 (60.5) | 0.438 |
| **Persistent CHI,** *n (%)* | 13 (52) | 15 (39.5) | 0.438 |
| **Birth weight [g],** *mean ± SD* | 3279 ± 807 (n=23) | 3039 ± 972 (n=37) | 0.925 |
| **SDS (Voigt),** *median (IQR)* | -0.9 (3.6) | -0.7 (2.5) | 0.919 |
| **Percentile (Voigt)** *median (IQR)* | 22 (76) (n=23) | 17 (93) (n=37) | 0.221 |
| **Gestational age [weeks],** *mean ± SD* | 39 ± 1 | 38 ± 2 | **0.002** |
| **Born abroad,** *n (%)* | 0 | 3 (7.9) | 0.270 |
| **Born via C-section,** *n (%)* | 13 (59.1)  (n= 22) | 28 (75.7)  (n=37) | 0.244 |
| **Risk factor for neonatal hypoglycemia,** *n (%)* | 10 (43.5)  (n=23) | 26 (70.3)  (n=37) | 0.058 |
| **Early onset CHI (< 30 days p.n.),** *n (%)* | 20 (80) | 36 (94.7) | 0.103 |
| **Delay between first symptoms and first BG measurement**,** *n (%)* | 6 (27.3)  (n=22) | 6 (18.2)  (n=33) | 0.517 |
| **Delay between first symptoms and first BG measurement [days],** *median (IQR)* | 61 (104.9) | 121.5 (203.8) | 0.481 |
| **Pancreatic surgery,** *n (%)* | 3 (12) | 2 (5.3) | 0.377 |
| **Hypoglycemic seizures,** *n (%)* | 10 (40) | 10 (26.3) | 0.281 |
| **Symptoms at onset of hypoglycemia,** *n (%)* | 17  (n=22) | 25  (n=33) | 1.0 |
| **Genetic CHI mutation,** *n (%)* | 6 (33)  (n=18) | 13 (56.5)  (n=23) | 0.209 |
| **Normal development,** *n (%)* | 14 (56) | 29 (76.3) | 0.104 |
| **Mildly abnormal neurodevelopment,** *n (%)* | 4 (16) | 6 (15.8) | 0.719 |
| **Severe brain injury,** *n (%)* | 7 (28) | 3 (18.4) | **0.039** |
| **First BG [mg/dl],** mean ± SD | 19.4 ± 13.3  (n=22) | 21 ± 13.6  (n=28) | 0.68 |
| **Lowest BG [mg/dl],** mean ± SD | 16.5 ± 10.2 | 18.2 ± 9.9  (n=35) | 0.516 |
| **Lowest BG < 20 mg/dl,** n (%) | 15 (60) | 22 (62.9)  (n=35) | 1.0 |
| **Insulin during hypoglycemia [mU/L],**  Median (IQR) | 9.3 (23)  (n = 24) | 15 (23)  (n = 36) | 0.179 |
| **Diazoxide-unresponsive,** n (%) | 3 (15)  (n = 20) | 6 (16.7)  (n = 36) | 1.0 |
| **Max. i.v. glucose infusion rate [mg/kg/min],** mean ± SD | 15.8 ± 4.1  (n=17) | 13.7 ± 3.3  (n=26) | 0.082 |
| **Insulin/glucose ratio [mU/l / mg/dl]**  mean ± SD | 1.9 ± 6.8  (n=22) | 2.1 ± 6.1  (n=31) | 0.915 |

* Comparison of normal development vs. severe brain injury only. Number (n), standard deviation (SD), percent (%), interquartile range (IQR), blood glucose (BG), congenital hyperinsulinism (CHI), postnatal (p.n.), N-values are only described in case of missing data. Bold type indicates significant result. **Delay between first symptoms and first BG measurements included patients who had clear symptoms of hypoglycemia but had a delay >12h between onset of symptoms and first BG measurement ***Lower-level hospital included basic care and specialty care nursery. High-level hospital included subspecialty care including neonatal intensive care unit (NICU) and regional perinatal center including NICU.

**Table 4: Patient characteristics of Germany-born patients only**

|  | **Total** | **Normal**  **development** | **Mildly abnormal**  **development** | **Severe brain injury** | **P-Value*** |
| --- | --- | --- | --- | --- | --- |
| **Number of patients,** *n (%)* | 78 | 55 (70.5) | 14 (18) | 9 (11.5) |  |
| **Age [years],** *median (IQR)* | 7 (8) | 6 (8) | 13 (10) | 5 (4) |  |
| **Female,** *n (%)* | 31 (39.7) | 22 (40) | 4 (28.6) | 5 (55.6) | 0.475 |
| **Transient CHI**, *n (%)* | 36 (46.2) | 31 (56.4) | 2 (14.3) | 3 (33.3) | 0.285 |
| **Persistent CHI**, *n (%)* | 42 (53.8) | 24 (43.6) | 12 (85.7) | 6 (66.6) | 0.285 |
| **Birth weight [g],** *mean ± SD* | 3132 ± 920  (n= 70) | 2882 ± 784 | 3940 ± 1054 | 3358 ± 713 | 0.115 |
| **Percentile (Voigt),** *median (IQR)* | 21 (84)  (n= 67) | 12 (43) | 97.5 (74) | 40.5 (79) | 0.141 |
| **SDS (Voigt),** *median (IQR)* | -0.8 (2.9) | -1.2 (1.8) | 2.1 (4.8) | -0.3 (3.2) | 0.141 |
| **Gestational age [weeks],** *mean ± SD* | 38 ± 2 | 38 ± 2 | 38 ± 2 | 39 ± 1 | 0.544 |
| **Born via C-section**, *n (%)* | 45 (70.3)  (n=64) | 32 (71.1)  (n=45) | 8 (66.7)  (n=12) | 5 (71.4)  (n=7) | 1.0 |
| **Risk factor for neonatal hypoglycemia**, *n (%)* | 39 (58.2)  (n=67) | 27 (57.4)  (n=47) | 10 (83.3)  (n=12) | 2 (25)  (n=8) | 0.131 |
| **Early onset CHI (< 30 days p.n.),** *n (%)* | 62 (79.5) | 43 (78.2) | 13 (92.9) | 6 (66.6) | 0.427 |
| **Delay between first symptoms and first BG measurement**, *n (%)* | 14 (20)  (n=70) | 7 (14.3)  (n=49) | 2 (16.7)  (n=12) | 5 (55.6) | **0.009** |
| **Delay between first symptoms and first BG measurement [days]**, *mean ± SD* | 61 (241)  (n = 70) | 119 ± 124 | 152 ± 128 | 74 ± 99 | 0.495 |
| **Pancreatic surgery,** *n (%)* | 6 (7.7) | 2 (3.6) | 2 (14.3) | 2 (22.2) | 0.092 |
| **Hypoglycemic seizures,** *n (%)* | 30 (38,5) | 19 (34.5) | 4 (28.6) | 7 (77.8) | **0.025** |
| **Symptoms at onset of hypoglycemia,** *n (%)* | 55 (78.6)  (n=70) | 36 (73.5)  (n=49) | 11 (91.7)  (n=12) | 8 (88.8) | 0.431 |
| **Genetic CHI mutation,** *n (%)* | 23 (43.4)  (n=53) | 12 (38.7)  (n=31) | 8 (57.1)  (n=14) | 3 (37.5)  (n=8) | 1.0 |
| **Birth at lower-level hospital**,** *n (%)* | 25 (41.7)  (n=60) | 14 (33.3)  (n=42) | 4 (44.4)  (n=9) | 7 (77.8) | **0.023** |

* Comparison of normal development vs. severe brain injury only. Number (n), standard deviation (SD), percent (%), interquartile range (IQR), blood glucose (BG), congenital hyperinsulinism (CHI), postnatal (p.n.). N-values are only described in case of missing data. Bold type indicates significant result. **Delay between first symptoms and first BG measurements included patients who had clear symptoms of hypoglycemia but had a delay >12h between onset of symptoms and first BG measurement ***Lower-level hospital included basic care and specialty care nursery. High-level hospital included subspecialty care including neonatal intensive care unit (NICU) and regional perinatal center including NICU.

**Table 5: Laboratory measurements and treatment of Germany-born patients only**

|  | **Total** | **Normal**  **development** | **Mildly abnormal**  **development** | **Severe brain injury** | **p-Value*** |
| --- | --- | --- | --- | --- | --- |
| **First BG [mg/dl],** *mean ± SD* | 21.6 ± 13.7  (n=59) | 22.9 ± 12.9  (n=39) | 23.7 ± 17.1  (n=11) | 13.2 ± 10.6  (n=9) | **0.034** |
| **Lowest BG [mg/dl],** *mean ± SD* | 19.2 ± 10.8  (n=74) | 20.8 ± 11.0  (n=52) | 17.5 ± 8.6  (n=13) | 12.1 ± 9.6  (n=9) | **0.031** |
| **Lowest BG < 20 mg/dl,** *n (%)* | 40 (54.1)  (n=74) | 24 (46.2)  (n=52) | 8 (61.5)  (n=13) | 8 (88.9)  (n=9) | **0.028** |
| **Insulin during hypoglycemia [mU/L],**  *Median (IQR)* | 12.8 (22)  (n=72) | 11.3 (20)  (n=49) | 16.3 (18)  (n=14) | 5.0 (43)  (n=9) | 0.653 |
| **Diazoxide-unresponsive**, *n (%)* | 11 (15.9)  (n=69) | 6 (12.2)  (n=49) | 4 (30.8)  (n=13) | 1 (14.3.3)  (n=7) | 1.0 |
| **Max. i.v. glucose infusion rate [mg/kg/min],** *mean ± SD* | 14.6 ± 3.7  (n=44) | 14.1 ± 4.0  (n=28) | 15.8 ± 3.1  (n=9) | 15.4 ± 3.6  (n=8) | 0.375 |
| **Insulin/glucose ratio [mU/l / mg/dl]**  *mean ± SD* | 1.7 ± 5.7  (n=65) | 1.3 ± 5.0  (n=42) | 1.0 ± 1.2  (n=14) | 4.8 ± 10.6  (n=9) | 0.780 |

* Comparison of normal development vs. severe brain injury only. Bold type indicates significant result. Number (n), standard deviation (SD), percent (%), interquartile range (IQR), blood glucose (BG), intravenous (i.v.)

**Table 6: Patient characteristics and laboratory measurements and treatment in patients born abroad**

|  | **Patients born aboard** |
| --- | --- |
| **Number of patients** | 9 |
| **Age [years],** median (IQR) | 8 (10) |
| **Female**, n (%) | 6 (66.7) |
| **Transient CHI**, n (%) | 1 (11.1) |
| **Persistent CHI**, n (%) | 8 (88.9) |
| **Birth weight [g],** mean ± SD | 4116 ± 1435  (n= 7) |
| **Percentile (Voigt),** median (IQR) | 95 (21)  (n=6) |
| **SDS (Voigt),** median (IQR) | 1.6 (2.6) |
| **Gestational age [weeks],** mean ± SD | 38 ± 2 |
| **Born via C-section**, n (%) | 3 (50)  (n=6) |
| **Risk factor for neonatal hypoglycemia,** n (%) | 4 (44.4) |
| **Early onset CHI (< 30 days p.n.),** n (%) | 6 (66.7) |
| **Delay between first symptoms and first BG measurement****, n (%) | 5 (55.6) |
| **Delay between first symptoms and first BG measurement [days],** median, IQR | 3 (454,4) |
| **Pancreatic surgery**, n (%) | 4 (44.4) |
| **Hypoglycemic seizures,** n (%) | 7 (77.8) |
| **Symptoms at onset of hypoglycemia**, n (%) | 7 (77.8) |
| **Genetic CHI mutation,** n (%) | 6 (75)  (n=8) |
| **Birth at lower-level hospital*,** n (%) | 0 (0)  (n=3) |
| **Normal neurodevelopment,** n (%) | 2 (22.2) |
| **Mildly abnormal neurodevelopment,** n (%) | 1 (11.1) |
| **Severe brain injury,** n (%) | 6 (66.7) |
| **First BG [mg/dl],** mean ± SD | 19.4 ± 15.1  (n=5) |
| **Lowest BG [mg/dl],** mean ± SD | 14.4 ± 8.0  (n=7) |
| **Lowest BG < 20 mg/dl,** n (%) | 5 (71.4)  (n=7) |
| **Insulin during hypoglycemia [mU/L],**  Median (IQR) | 11.9 (29)  (n=7) |
| **Diazoxide-unresponsive,** n (%) | 3 (37.5)  (n=8) |
| **Max. i.v. glucose infusion rate [mg/kg/min],** mean ± SD | 11.0 ± 1.4  (n=2) |
| **Insulin/glucose ratio [mU/l / mg/dl]**  mean ± SD | 0.76 ± 1.0  (n=7) |

Number (n), standard deviation (SD), percent (%), interquartile range (IQR), blood glucose (BG), congenital hyperinsulinism (CHI), postnatal (p.n.). N-values are only described in case of missing data. *Delay between first symptoms and first BG measurements included patients who had clear symptoms of hypoglycemia but had a delay >12h between onset of symptoms and first BG measurement **Lower-level hospital included basic care and specialty care nursery. High-level hospital included subspecialty care including neonatal intensive care unit (NICU) and regional perinatal center including NICU. Comparative analysis was not performed due the small number of patients in the subgroup.

**Hypoglycemia Questionnaire**

| **Age:** | _____________________________ | **Weight:** | _____________________________ |
| --- | --- | --- | --- |
| **Sex:** | 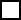 Female 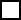 Male | **Height:** | _____________________________ |

**Place of Birth:**
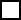
 university hospital


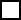
 large hospital with ICU (intensive care unit) for children


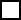
 small local hospital without ICU for children


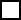
 home birth

If the child was born in a hospital, was there a pediatric department in the hospital?
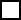
 Yes
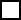
 No

**Which form of Hypoglycemia did/ does your child have?**


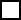
 Hyperinsulinism (congenital disease with high release of insulin and severe hypoglycemia)


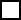
 Transient hypoglycemia (hypoglycemia longer than ten days of life, but not longer than twelve months)


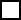
 Transient hypoglycemia (hypoglycemia after the third day of life, but not longer than ten days)


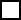
 Other forms: ____________

**Does your child have any other illnesses?**
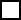
 Yes
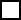
 No

**If yes, please state the illnesses and the age of the diagnoses: _______________________________________**

**___________________________________________________________________________________________________**

**Current medication:** _____________________________________________________________________

_____________________________________________________________________

**Pregnancy and Birth:**

*(The following section asks questions about your childbirth and the course of pregnancy. In order to answer the questions as accurately as possible, it is helpful if you take the yellow examination booklet of your child at hand.)*

**Were there any complications during pregnancy or birth?**
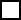
 Yes
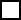
 No

Were there complications…

… with the child? What kind of complication? _____________________________________________________

... with the mother? What kind of complication? ___________________________________________________

Other complications: _____________________________________________________________________________

1. **Was your child a pre-term delivery?**
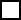
 Yes
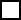
 No

1. **Was your child a post-term delivery?**
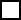
 Yes
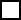
 No

1. **In which week of pregnancy was your child delivered?** _____________________________________
2. **Birthweight:** ______________ (g) **Length at birth:** _____________ (cm)

**Head circumference at birth:** ______________ (cm) **Apgar-Score at birth:** _____/______/______

1. **How was the child delivered?**
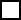
 Planned C-Section
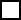
 Vaginal Delivery


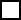
 Emergency C-Section
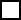
 Vacuum Extractor


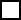
 Forceps delivery
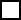
 Other: _____________

1. **Did the mother have gestation diabetes?**
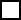
 Yes
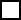
 No

If yes, how was it treated? Diet
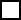
 Insulin
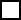
 Other: ____________________________

1. **Were there any complications with your child after birth?**
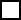
 Yes
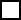
 No

If yes, which?

| 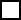 blood poisoning (sepsis) | 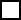 suffocation (asphyxia) / respiratory distress |
| --- | --- |
| 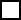 infection | 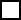 respiratory problems (respiratory adjustment  disorder) |
| 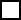 cerebral hemorrhage | 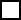 cold body temperature (hypothermia) |
| 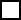 newborn-jaundice (hyperbilirubinemia) | |
| 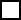 Other: ______________________________________ | |

1. **When was the child (breast) fed for the** **first time?**


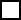
 directly after birth
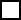
 within 30 Min
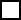
 after 1h
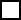
 after 2h
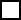
 after 3h

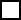
 >4h
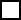
 The child was not breastfed
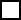
 Other: ____________________________

1. **How long did your child stay in the hospital after birth?** Days: _________________________

1. **Was there a transfer to another hospital or ward?**
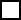
 Yes
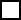
 No

If yes, whereto and when?

_____________________________________________________________

What was the reason for the transfer?

_____________________________________________________________

**Family History**

*(Questions about your family history are asked below to assess whether there is a risk of hypoglycemia in your family.)*

1. **Was the affected child your firstborn?** no
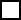
 yes
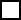


How many children do you have? : __________________________

1. **Do any family members suffer from Diabetes?** no
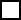
 yes
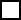


If yes, who?

_________________________________________________________________________________

1. **Do or did any family members suffer from low**

**blood glucose (Hypoglycemia)?** no
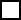
 yes
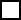


If yes, who?

_________________________________________________________________________________

1. **Do any of your other children have any illnesses** no
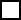
 yes
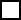


**or preexisting conditions?**

If yes, who (number of child) and which disease? _________________________________________

1. **Do you as parents have any illnesses or** no
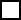
 yes
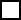


**preexisting conditions?**

If yes, who and what? _________________________________________________________________

1. **Is there any parental genetic relationship?** no
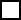
 yes
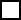


If yes, which parental genetic relationship? ______________________________________________

**Symptoms**

*(The following section deals with symptoms that your child may have experienced during hypoglycemia. Our goal is to find out whether we can identify certain symptoms as red flags that allow us to distinguish between severe hypoglycemia due to hyperinsulinism and other forms of hypoglycemia in newborns.)*

| 1) **Were there any symptoms that led to the diagnosis of hypoglycemia?**  **If yes, which symptoms occurred?** | no | 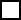 | yes | 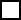 |
| --- | --- | --- | --- | --- |

| - tiredness, exhaustion - poor feeding - loss of consciousness, coma - seizures - hyperreflexia - shrill cries | - floppy muscles - headache, dizziness - nausea/vomiting - pale skin, blue lips - agitation, sweating - respiratory problems - other: ________________________________ |
| --- | --- |

1. **When did symptoms occur for the first time?**  *(time after birth)*

<12h
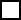
 12-24h
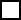


24-48h
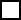
 48-72h
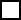


3^rd^ -7^th^ Day of life
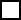
 > 7^th^ Day of life
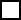


1. **Who noticed the symptoms?**

Parents
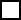
 Physician
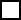


Nurses
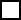
 Midwife
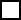


Other: _________________________________

1. **In which order did the symptoms occur?** *(Please describe with time specification)*

____________________________________________________________________________________________

1. **At which blood glucose level have you noticed symptoms in your child for the first time?**

value: ________________________ (mg/dl or mmol/l)

1. **Were there any symptoms of hypoglycemia at a later stage?**

| **If yes, which symptoms occurred?** |  |  |  |
| --- | --- | --- | --- |

| - tiredness, exhaustion - poor feeding - loss of consciousness, coma - seizures - hyperreflexia - shrill cries | - floppy muscles - headache, dizziness - nausea/vomiting - pale skin, blue lips - agitation, sweating - respiratory problems - other: ________________________________ |
| --- | --- |

1. **At which blood glucose** **level have you noticed symptoms in your child at later times?**

value: ________________________ (mg/dl o. mmol/l)

1. **Did your child ever have severe symptomatic hypoglycemia? (i.e. severe disturbance of**

**consciousness, coma or seizures in low blood glucose)** No
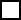
 Yes
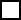


**If yes, when did they occur?** *(DoL: Day of Life)*

I do not know
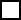


| 1^st^ DoL  2^nd^ -4^th^ week | 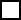  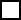 | 2^nd^ DoL  2^nd^-3^rd^ month | 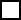  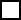 | 3^rd^ DoL  > 3^rd^ month | 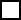  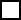 | 3^rd^ -7^th^ DoL 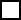  other: ________________ |
| --- | --- | --- | --- | --- | --- | --- |

9) **Please state the lowest glucose level while your child had symptomatic hypoglycemia?**

value: _________________________ mg/dl o. mmol/l

**Clinical Diagnostics**

*(The following section asks questions about blood glucose levels and other tests related to your child's hypoglycemia, especially during the first few days of life. The more accurate the values, the better we can use them later in the evaluation to give recommendations on blood glucose control and how to deal with hypoglycemia. You may have the doctor's letters at home that tell you your blood glucose levels.)*

1. **When was the blood glucose level first controlled after birth?**

< 30 mins.
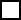
 1-2h
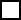
 3-6h
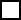
 6-12h
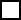


12-24h
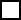
 24-48h
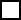
 48-72h
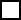
 other: ________________

1. **Why was your child's blood glucose level controlled?**

Symptoms occurred
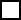
 There was a risk of hypoglycemia (e.g.
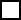


premature birth, light, heavy child at birth, maternal diabetes, complications at birth/stress)

I don't know
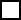
 A routine sugar check was performed
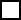


1. **How high was the blood glucose level at the first measurement?**

>70mg/dL
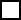
 60-70mg/dL
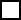
 50-60mg/dL
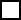
 45-50mg/dL
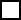


35-45mg/dL
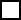
 25-35mg/dL <25mg/dL exact value: __________

1. **How often was the blood glucose level controlled after the first measurement?**

every 30 min. hourly every 2h every 3h

every 4-6h >6h There were other: ________________

no controls

1. **How long did it take to get the first normal blood glucose level > 70mg / dl after the first hypoglycemia?**

30 min. 1 h 2 hrs 3 hrs

4-5 hrs 6-8 hrs >8 hrs value: _________________

1. **What was the lowest blood glucose level ever measured?**

>70mg/dL 60-70mg/dL 50-60mg/dL 45-50mg/dL

35-45mg/dL 25-35mg/dL <25mg/dL exact value: ___________

1. **When was the lowest blood glucose level measured?** *(DoL: Day of Life)*

1^st^ DoL 2^nd^ DoL 3^rd^ DoL 3^rd^ -7^th^ DoL

2^nd^ -4^th^ week 2^nd^-3^rd^ month > 3^rd^month other: ________________

8) **Did your child participate in a blood** **glucose screening without prior symptoms or suspicion of a low** **blood glucose level?**

no yes

**If yes, why was the screening conducted?**

- There was a risk for low blood glucose levels What risk? _______________

- all infants are being screened in the hospital

- other cause: ____________________________________________________________________

**If yes, when was the screening conducted?** (*time after birth)*

| < 1h 1-2h 2-3h  6-24h 2^nd^-3^rd^ DoL >3^rd^ DoL      9) **Were there any other examinations/tests to find the cause of low blood glucose levels?** |  | 3-6h other: ________________      no yes |
| --- | --- | --- |

**If yes, which examinations/tests?**

*(mark all that apply)*

blood test urine test fasting-test

ultrasound MRI PET-CT genetic testing

other: ___________________________________________________

| 10) **What was the reason for further diagnostics (examination)?** |  |
| --- | --- |
| Severe hypoglycemia Missing therapy response  Frequent hypoglycemia Long duration of hypoglycemia (>3. DoL)  Symptoms Siblings suffer from hypoglycemia  Suspicion of syndromic Suspicion of other underlying diseases  disease      11) **Has a blood sample been taken during hypoglycemia?** no yes | |

If so, how high was the insulin value in the examination? ____________________ (mU/l)

12) **Was it possible to detect the cause of low blood glucose levels?**

no yes

**If yes, what was the cause?** ___________________________________________________________________

**How old was your child at the time of diagnosis?** age: _____________________

**In hyperinsulinism: Has the genetic cause been** no yes  **determined?**

If so, which disease-causing mutation is present? ________________________

e.g. ABCC8 (paternal or maternal inherited, homozygous, heterozygous), KCNJ11(paternal or maternal inherited, homozygous, heterozygous), GCK, GLUD1, HADH, HNF4A, UCP2, SLC16A1 (MCT1), HK1, PMM2, MPI, PGM1, INSR, others

If yes, is there is a focal form or a diffuse form of hyperinsulinism?

**When was the first time a physician discussed with you that the hypoglycemia of your child are not normal?** ______________________

**When was the first time a physician discussed with you that there is a suspicion of hyperinsulinism/a permanent hypoglycemia?** __________________________

13) **When did your physician decide to conduct further clinical diagnostic?** *(DoL: Day of Life)*

| 1^st^ DOL  2^nd^-4^th^ week |  | 2^nd^ DOL  2^nd^-3^rd^ month |  | 3^rd^ DOL  > 3^rd^ month |  | 3^rd^-7^th^ DOL  other: ________________ |
| --- | --- | --- | --- | --- | --- | --- |

**What was the reason for those further investigations?**

| very low blood glucose levels  frequent hypoglycemia symptoms  suspected syndromal disease  no response to therapy | hypoglycemia beyond the 3^rd^ day of life  siblings also had hypoglycemia  suspicion of other underlying cause  other: ___________________________________________ |
| --- | --- |

1. **How long was the time frame between initial hypoglycemia and diagnosis?**

| < 3 days  10-14 days |  | 3-5 days  2-3 weeks |  | 5-7 days  > 4 weeks |  | 7-10 days  other: ________________ |
| --- | --- | --- | --- | --- | --- | --- |

1. **When did your child have a normal blood glucose level for the first time?** (*time after birth)*

| <12h  3^rd^–5^th^ DoL |  | 12-24h  5^th^–7^th^ DoL |  | 24-48h  7^th^–14^th^ DoL |  | 3^rd^ DoL  other: ________________ |
| --- | --- | --- | --- | --- | --- | --- |

**Therapy of first hypoglycemia**

*(The following section deals with the therapy that was performed in the hospital after your child was diagnosed with hypoglycemia for the first time. The aim is to find out whether the effect of the therapy can better or earlier distinguish between children with severe hypoglycemia due to hyperinsulinism and children with normal hypoglycemia. We are aware that it is difficult to answer these questions. If you can't remember well or are unsure, you can tick "I'm not sure")*

1. **What was the initial treatment after the first measurement of low blood glucose?**

Nothing/Waiting more frequent feeding Dextrose gel

Glucose orally Glucose Infusion Glucagon

Diazoxid I’m not sure Other: ________________________

1. **Did the blood glucose levels normalize during the initial** no yes  **treatment?**

If yes, was the treatment continued? no yes

Did blood glucose levels increase to a normal value? no yes

What was the highest blood glucose under the 1^st^ treatment?: ___________(mg/dL) (approx.)

What was the lowest blood glucose under the 1^st^ treatment?: ___________(mg/dL) (approx.)

1. **How long did your child receive the first initial treatment?**

< 6h 6-12h 12-24h 2-3 days

3-5 days 5-7 days 7-14 days other: _________________

1. **How often did further hypoglycemia occur under the initial treatment?**

Several times 1x per day Every 2-3 days 1x per week

few times Not at all Other:______________________

I don't remember

1. **Was there a change of treatment?** no yes

**Why was the treatment changed?**

Blood glucose levels didn’t change under the 1^st^ treatment

Blood glucose levels decreased further under the 1^st^ treatment

Despite an increase, the blood glucose levels didn’t normalize

There were adverse effects

Symptoms (still) occurred

The cause of disease was identified and a long-term treatment started

other: __________________________________________________________________

1. **What was the second treatment after the first measurement of low blood glucose?**

Nothing/Waiting more frequent feeding Dextrose gel

Glucose orally Glucose Infusion Glucagon

Diazoxid I’m not sure Other: ________________________

7) **Did the blood glucose levels rise during the second treatment?** no yes

If yes, was the treatment continued? no yes

Did blood glucose levels rise to a normal value? no yes

What was the highest blood glucose under the 2^nd^ treatment?: ______________(mg/dL)

(approx.)

What was the lowest blood glucose under the 2^nd^ treatment?: _______________(mg/dL) (approx.)

1. **How long did your child receive the second treatment?**

< 6h 6-12h 12-24h 2-3 days

3-5 days 5-7 days 7-14 days

1. **How often did further hypoglycemia occur under the second treatment?**

Several times 1x per day Every 2-3 days 1x per week

few times Not at all Other:______________________

I don't remember

1. **Was there another change of treatment?** no yes

**Why was the treatment changed?**

Blood glucose levels didn’t change under the 2^st^ treatment

Blood glucose levels decreased further under the 2^st^ treatment

Despite an increase, the blood glucose levels didn’t normalize

There were adverse effects

Symptoms (still) occurred

The cause of disease was identified and a long-term treatment started

1. **Were there any more therapeutic attempts to normalize** no yes

**blood glucose levels? (3 or more)**

If yes, how many attempts? _______________________________________________________________

| 12) **Which other treatments were attempted?** | *(mark all that apply)* |
| --- | --- |
| Glucose Infusion more frequent feeding  Glucose orally carbohydrate-rich diet  Diazoxid I’m not sure | Dextrose Gel Glucagon other: ____________ |

1. **What was the lowest ever measured blood glucose level after initiation of treatment?**

| >70mg/dL  35-45mg/dL |  | 60-70mg/dL  25-35mg/dL |  | 50-60mg/dL  <25mg/dL |  | 45-50mg/dL  exact value: __________ |
| --- | --- | --- | --- | --- | --- | --- |

1. **If your child received a glucose infusion, what was the maximum need (infusion rate) to stabilize blood sugar?** (*maybe you'll find the information in a doctor's letter)* Pass rate: ________________________ (e.g. X mg/kg/min)

1. **Was it possible to terminate the treatment after normalization of blood glucose levels?**

| Yes, the treatment was stopped  No, a long-term treatment was started |  | age at termination of treatment:  __________________________________ |
| --- | --- | --- |

**Long-Term Treatment**

*(The following section deals with the topic of long-term therapy. Please state therapeutic measures that are used to permanently adjust the blood sugar level in the case of persistent hypoglycemia in order to release the child home despite hypoglycemia. Medications that are used for long-term therapy include diazoxide, lanreotide, ocretide, sirolimus and glucagon. Also continuous probes, i.e. a stomach probe or a nourishing therapy, can be defined as continuous therapy.)*

1. **How old was your child at the initiation of a long-term treatment?** age: _____________________

1. **How long was the time period between initial hypoglycemia and initiation of a long-term treatment?**

<3 days 3-7 days 7—14 days 2-4 weeks 4-8 weeks

>2 month other: _________________________

**What was the first long-term treatment?**

Diazoxid Nasal tube Sirolimus

feeding

Lanreotid Diet Glucagon

Ocreotid I’m not sure other: ________________

**Period of therapy:**___________________________________________ (e.g. 1-6 month of life)

**Duration of therapy**: ___________________________ (Months or years)

1. **Was the treatment effective?** no yes

If not, why was it ineffective? ______________________________________________________________

4) **Were there any side effects?** no yes

If yes, which adverse effects? _____________________________________________________________

1. **Did further hypoglycemia occur during therapy?** no yes

If yes, how often?

| Daily several times a week approx. once a week |
| --- |
| approx. every 2 weeks Approx. every 3-4 weeks rarely |

In which range were the lowest blood glucose levels during the long-term therapy?

>70mg/dL 60-70mg/dL 50-60mg/dL 45-50mg/dL

35-45mg/dL 25-35mg/dL 15-25mg/dL <15mg/dl

I don’t remember exact value: ________________________________

1. **Did your physician try any other long-term treatment?** no yes

If yes, please mark all that apply:

Diazoxid Nasal tube Sirolimus

feeding

Lanreotid Diet Glucagon

Ocreotid I’m not sure other: ________________

1. **Did your child have further hypoglycemia under** no yes

**the long term treatment?**

If yes, how often? ***______________________*** Lowest value? ________________________________

**Was it possible to terminate the long-term**  no yes **treatment after normalization of blood glucose levels?**

child’s age at termination of treatment? age: _________________________________________

**Surgical Treatment**

1. **Did your child have pancreatic surgery?** no yes

If yes, what kind of surgery? _____________________________________________________________

1. **Does your child have any of these illnesses?** *(mark all that apply and state age at diagnosis)* Diabetes Mellitus age: ________ Enzyme Deficiency age: _________

**Outcome**

*(The following section deals with the question of whether your child may have suffered brain damage as a result of hypoglycemia. It is about changes that are usually not found in healthy children and, for example, show significant differences compared to siblings.*

1. **Has your child attended a kindergarten/daycare or is this currently the case?** no yes

1. **What kind of school does your child go to?**

| Kindergarden  High School |  | Pre-School  Special School |  | Elementary School  other: ______________________ |
| --- | --- | --- | --- | --- |

**Was your child regularly enrolled?** no yes at what age?: ________

**Which school grade is your child currently attending?** grade: _____________________________

**Does an integration assistance accompany your child at school?** no yes If yes,why? *(keywords) ):* ____________________________________________________

1. **Were all milestones of development achieved on time?** no yes

Which milestones were achieved lately? At what age were they reached? _________________

__________________________________________________________________________________________

**Motorics/fine motorics:**

*Movements controlled yes, at the age of:*

| crawling | 􀀀 no 􀀀 yes _______________ |
| --- | --- |
| Free running | 􀀀 no 􀀀 yes _______________ |
| Tricycling / Trampling tractor/ Running wheel | 􀀀 no 􀀀 yes _______________ |
| Cycling without support wheels | 􀀀 no 􀀀 yes _______________ |
| Handling scissors (cutting) | 􀀀 no 􀀀 yes _______________ |
| Open and close buttons | 􀀀 no 􀀀 yes _______________ |
| Which hand does your child use as a priority? | 􀀀 right 􀀀 left |
|  |  |

**Language – Sehen**

*Basic skills mastered yes, at the age of:*

| Babbling, first sounds | 􀀀 no 􀀀 yes _______________ |
| --- | --- |
| First words | 􀀀 no 􀀀 yes _______________ |
| 2-3 word sentences | 􀀀 no 􀀀 yes _______________ |
| Complete sentences | 􀀀 no 􀀀 yes _______________ |

| 4) | **Does your child have any developmental** no **delay?**  Motor Function Language Skills  Cognition Mental  Retardation |  | *(or mark all that apply)*  Social Development  other: _______________________ |
| --- | --- | --- | --- |

When was the developmental delay first diagnosed? Child’s age: ____________________

5) **Did or does your child regularly receive special support ?** no yes

**If yes, tick the appropriate**

Physiotherapy Speech therapy Ergotherapy

Early Childhood Therapy Psychotherapy School support

other: ________________________________________________________

6) **Does your child have any neurological disorders?** No *(or mark all that apply)*

Epilepsy/Seizures Cerebral Palsy

Blindness Deafness

other: ____________________________________________________

When was the disorder diagnosed? child’s age at diagnosis: ____________________

7) **Did your child ever participate in an IQ-Test or developmental testing?** no yes

If yes, what was the result? ________________________________________________________

| 8) | **Was there any imaging of your child’s brain? (MRI, CT)** If yes, were there any abnormal findings? | no  no |  | yes  yes |  |
| --- | --- | --- | --- | --- | --- |

If yes, were there anomalies and what kind of anomalies?__________________________________________
